# Erythrocyte ω-3 polyunsaturated fatty acids are inversely associated with the risk of oral cancer: a case-control study

**DOI:** 10.1038/s41387-020-00140-1

**Published:** 2020-09-28

**Authors:** Qing Chen, Jing Wang, Jing Wang, Jing Lin, Lin Chen, Li-song Lin, Li-zhen Pan, Bin Shi, Yu Qiu, Xiao-yan Zheng, Fa Chen, Bao-chang He, Feng-qiong Liu

**Affiliations:** 1 Department of Epidemiology and Health Statistics, Fujian Provincial Key Laboratory of Environment Factors and Cancer, School of Public Health, Fujian Medical University, Fuzhou, China; 2 Key Laboratory of Ministry of Education for Gastrointestinal Cancer, Fujian Medical University, Fuzhou, China; 3 Laboratory Center, School of Public Health, Fujian Medical University, Fuzhou, China; 4 Department of Oral and Maxillofacial Surgery, the First Affiliated Hospital of Fujian Medical University, Fuzhou, China

**Keywords:** Cancer, Risk factors

## Abstract

**Objectives:**

Evidence about ω-3 polyunsaturated fatty acids (ω-3 PUFAs) and oral cancer risk were limited. We aimed to evaluate the association of erythrocyte ω-3 PUFAs with the risk of oral cancer in a population from China.

**Methods:**

Erythrocyte ω-3 PUFAs of 236 oral cancer patients and 300 controls were determined by gas chromatography. Restricted cubic spline and logistic regression were used to analyze the association between erythrocyte ω-3 PUFAs and oral cancer risk. The crude and adjusted OR with 95% CI was calculated. Stratification analysis was performed to explore the potential interaction between ω-3 PUFAs and other traditional risk factors such as smoking and drinking.

**Results:**

Eicosapentaenoic acids (EPA), docosahexaenoic acids (DHA) and ω-3 index were negatively but non-linearly related to risk of oral cancer as observed by restricted cubic spline. The adjusted *OR* of EPA, DHA, and ω-3 index were 0.52 (95% *CI*: 0.35–0.76), 0.19 (95% *CI*: 0.08–0.44), 0.20 (95% *CI:* 0.09–0.44), respectively. Stratification analysis showed that the adverse correlation between EPA and oral cancer was only significant in the non-smoking group, while the adverse correlation of ɑ-linolenic acid (ALA), EPA, and DHA were only significant in the non-drinking group. General multiplicative interactions were observed between ω-3 PUFAs and smoking or drinking.

**Conclusions:**

Adverse but non-linear associations were observed between erythrocyte EPA, DHA, ω-3 index, and oral cancer risk. Additionally, there were multiplicative interactions between ω-3 PUFAs and other behavior factors such as smoking and drinking. The protective effect of ω-3 PUFAs maybe more significant in the non-smoking or non-drinking population.

## Introduction

Oral cancer is one of the most common malignant tumors of the head and neck cancer (HNC), with an increasing incidence globally. There are 354,864 new cancer cases and 177,384 deaths per year worldwide with higher rates in developing countries^1,2^. It has been a major public health problem in less developed countries. The recognized etiologic factors of oral cancer include age, sex, smoking, drinking^3^, HPV^4^, consumption of betel nut^5^, etc. However, in our previous studies, we observed that a large proportion (about 50%) of oral cancer patients were not exposed to the above behavior risk factors, indicating that there are other unrevealed important factors. In the past decades, increasing interest has arisen regarding to the relation between lipid metabolism, especially fatty acids, and carcinogenesis.

Erythrocyte membrane fatty acids are objective and stable biomarkers of fatty acids exposure. Compared with serum or plasma fatty acid, erythrocyte membrane fatty acid can reflect the medium to long-term fatty acid exposure status of human body. Erythrocyte membrane fatty acid composition is determined by a combination of fatty acid diet intake and metabolism. All major classes of fatty acids can be found in erythrocyte, which include saturated, monounsaturated, trans unsaturated, and polyunsaturated (both ω-3 and ω-6 families). Among all the major classes of fatty acids, ω-3 PUFAs have got the most research interest. ω-3 PUFAs include ɑ-linolenic acid (ALA, 18:3 n-3), eicosapentaenoicacids (EPA, 20:5 n-3), docosapentaenoic acids (DPA, C22:5 n-3), docosahexaenoic acids (DHA, 22:6 n-3), and ω-3 index^6^. Reduced ω-3 PUFAs in erythrocyte membrane have been reported to be associated with higher coronary heart disease risk^7–9^ and could also increase risk of neuropsychiatric^10^. In addition, increasing evidence suggest that dietary or blood ω-3 PUFAs are associated with a variety of cancers, such as HNC, esophageal cancer^11^, colorectal cancer^12,13^, and breast cancer^14,15^. Moreover, those observed associations are largely independent of other known risk factors.

Nevertheless, the role of dietary and nutritional factor in the etiology of oral cancer has been largely neglected, and there has been very limited evidence about the association between ω-3 PUFAs and oral cancer. To clarify the potential association may help to elucidate other underlying etiology of oral cancer which cannot be explained by revealed risk factors. Therefore, we conducted a case control study: (1) to assess the association between erythrocyte ω-3 PUFAs and risk of oral cancer, (2) to evaluate if there are interactions between erythrocyte ω-3 PUFAs and other reported risk factors, such as smoking and drinking in oral cancer.

## Subjects and methods

### Study population and data collection

A hospital-based case-control study was conducted with patients who were recruited from the First Affiliated Hospital of Fujian Medical University from September 2010 to January 2019 in Fujian province, China. The inclusion criteria was as follows: (1) histologically confirmed primary oral cancer; (2) Chinese population with residence in Fujian Province at least for 10 years; and (3) age between 20 and 80 years old; Patients with recurrent or metastasized cancer, with previous chemotherapy or radiotherapy treatment were excluded. Control participants were recruited from the health examination center of the same hospital during the same period. Current or past experience of cancer was excluded. Finally, 236 patients and 300 control participants were admitted to the study.

All participants provided a signed informed consent. The study protocol was approved by the Institutional Review Board of Fujian Medical University (Approval number: 2011053; Approval date: March 10, 2011) and conducted in accordance with the ethical standards described in the Declaration of Helsinki. Information of demographic characteristics were collected using a structured questionnaire for both case and control group by face to face interview.

### Blood collection

Approximately 5 mL fasting blood sample was collected for each participants in an EDTA tube. All the blood samples of oral cancer patient were obtained the second day after patients were admitted to the hospital, to insure that the exposure detected was prior to any drug treatment or examination. After collection, the blood samples were centrifuged at 1500 rpm for 10 min at 4 °C to separate the erythrocytes.

### Erythrocyte membrane fatty acids

Erythrocytes were haemolysed in hypotonic Tris–HCl buffer, pH = 7.4, at 4 °C for 2 h. A membrane pellet was obtained by ultra centrifugation (12,000 rpm for 30 min at 4 °C) from which lipids were extracted with chloroform/methanol (2:1, v/v), and the extract was dried in N2(ref. ^16^). Fatty acids methyl esters (FAME) were obtained by incubating with 14% boron trifluoride ether/methanol (1:3,v/v) solution at 60 °C for 10 h. FAME was extracted into 1 ml hexane, vaporized to dryness and redissolved in hexane for gas chromatography analysis^17^.

FAME were analyzed using an Agilent 7890B gas chromatograph (Agilent, Palo Alto, CA, USA) fitted with flame ionization and DB-23 fame column (60 m × 0.25 mm inside diameter, film thickness of 0.15 μm, Agilent, Palo Alto, CA, USA). The carrier gas was nitrogen and the inlet worked in a split model with a split ratio of 10:1. The injection temperature was 250 °C and detection temperature was 280 °C. The starting temperature of the column was 120 °C. After 5 min, the temperature was programmed from 120 to 175 °C at a rate of 10 °C/min, the temperature was continuously increased 5 °C/min up to 210 °C and finally up to 230 °C.

Individual fatty acids was identified by comparison with known standards (Sigma-Aldrich Inc., USA and AccuStandard Inc., USA), and expressed as a percentage of total fatty acids quantified from peak areas. The coefficients of variation for ALA, EPA, DPA, and DHA were 1.03%, 0.77%, 1.24%, 1.32%, respectively.

### Expression of erythrocyte membrane ω-3 PUFAs

Four individual fatty acids, ALA, EPA, DPA, DHA were included in the study and expressed as percentages of total fatty acids. Total ω-3 PUFAs refers to ALA + EPA + DPA + DHA and ω-3 index refers to EPA + DHA^18^.

### Statistical analysis

Distribution of demographic characteristic between case and control were examined using *χ*^2^ test or Fisher’s exact test. For the comparison of continuous variables between the two groups, *t*-test was performed for normally distributed data, while Wilcoxon rank sum test was adopted for data with skewed distribution. Restricted cubic spline was used to reveal the potential non-linear relationship between erythrocyte ω-3 PUFAs and risk of oral cancer. Then erythrocyte ω-3 PUFAs were included in unconditional logistic regression model as a continuous variable or as a dichotomous variable categorized according to the median of control group. The crude and adjusted OR with 95% CI was calculated to evaluate the effect of erythrocyte ω-3 PUFAs on oral cancer risk. All of the data were analyzed using Stata software version 13.1. All *P*-values were two-sided and *P* < 0.05 was considered as statistically significant.

## Results

A total of 236 patients and 300 control participants were included in the study. Details of demographic characteristics of all participants are listed in Table 1. The median age of the patient and control group was 59 and 66 years, respectively. Higher proportion of male, smoking and drinking exposure, lower BMI were observed in the cases group compared with controls. The distribution of ethnic group, marital status, origin, family history of cancer and occupation were similar between the case and control group. Univariate analysis was also performed to establish the risk factors associated with oral cancer. The results showed that sex, age, education, BMI, oral hygiene, smoking and drinking were associated with risk of oral cancer. Variables with a significant association were included in the multivariate model in subsequent analysis.

**Table1:** Main characteristics of case and control subjects.

Variable	Control (%) (*n* = 300)	Case (%) (*n* = 236)	*χ*^2^	*P* value	Univariable OR(95% C*I*)
Sex			9.01	<0.01	
Male	110 (36.67)	117 (49.58)			1.00
Female	190 (63.33)	119 (50.42)			0.59 (0.42–0.83)
Age (year)	66 (64–70)	59 (49–67)	–	<0.01^a^	0.92 (0.91–0.94)
Education level			21.89	<0.01	
Illiterate	51 (17.00)	12 (5.08)			1.00
Primary and middle school	194 (64.67)	157 (66.53)			3.44 (1.77–6.68)
High school and above	55 (18.33)	67 (28.39)			5.18 (2.51–10.67)
Ethnic group			–	0.19^b^	
Han nationality	300 (100.00)	234 (99.15)			–
Other	0 (0.00)	2 (0.85)			–
Marital status			2.75	0.1	
Married	275 (91.67)	206 (87.29)			1.00
Single	25 (8.33)	30 (12.71)			1.60 (0.91–2.81)
Origin			0.18	0.67	
Rural area	172 (57.33)	131 (55.51)			1.00
Urban area	128 (42.67)	105 (44.49)			1.08 (0.76–1.52)
Family history of cancer			3.28	0.07	
No	274 (91.33)	204 (86.44)			1.00
Yes	26 (8.67)	32 (13.56)			1.65 (0.96–2.86)
BMI (kg/m^2^)			16.98	<0.01	
18.5–23.9	149 (49.67)	136 (57.63)			1.00
<18.5	11 (3.67)	24 (10.17)			2.39 (1.13–5.06)
≥24	140 (46.67)	76 (32.20)			0.59 (0.41–0.86)
Occupation			1.78	0.41	
Farmer	121 (40.33)	82 (34.75)			1.00
Worker	36 (12.00)	32 (13.56)			1.31 (0.75–2.28)
Other	143 (47.67)	122 (51.69)			1.26 (0.87–1.82)
Oral hygiene			10.00	<0.01	
Well	31 (10.33)	42 (17.80)			1.00
Moderate	168 (56.00)	138 (58.47)			0.61 (0.36–1.02)
Poor	101 (33.67)	56 (23.73)			0.41 (0.23–0.72)
Smoking status			17.13	<0.01	
No	241 (80.33)	152 (64.41)			1.00
Yes	59 (19.67)	84 (35.59)			2.26 (1.53–3.33)
Alcohol consumption			12.72	<0.01	
No	259 (86.33)	175 (74.15)			1.00
Yes	41 (13.67)	61 (25.85)			2.20 (1.42–3.42)

^a^Median(inter quartile range).

^b^Fisher’s exact test.

**Fig. 1:**
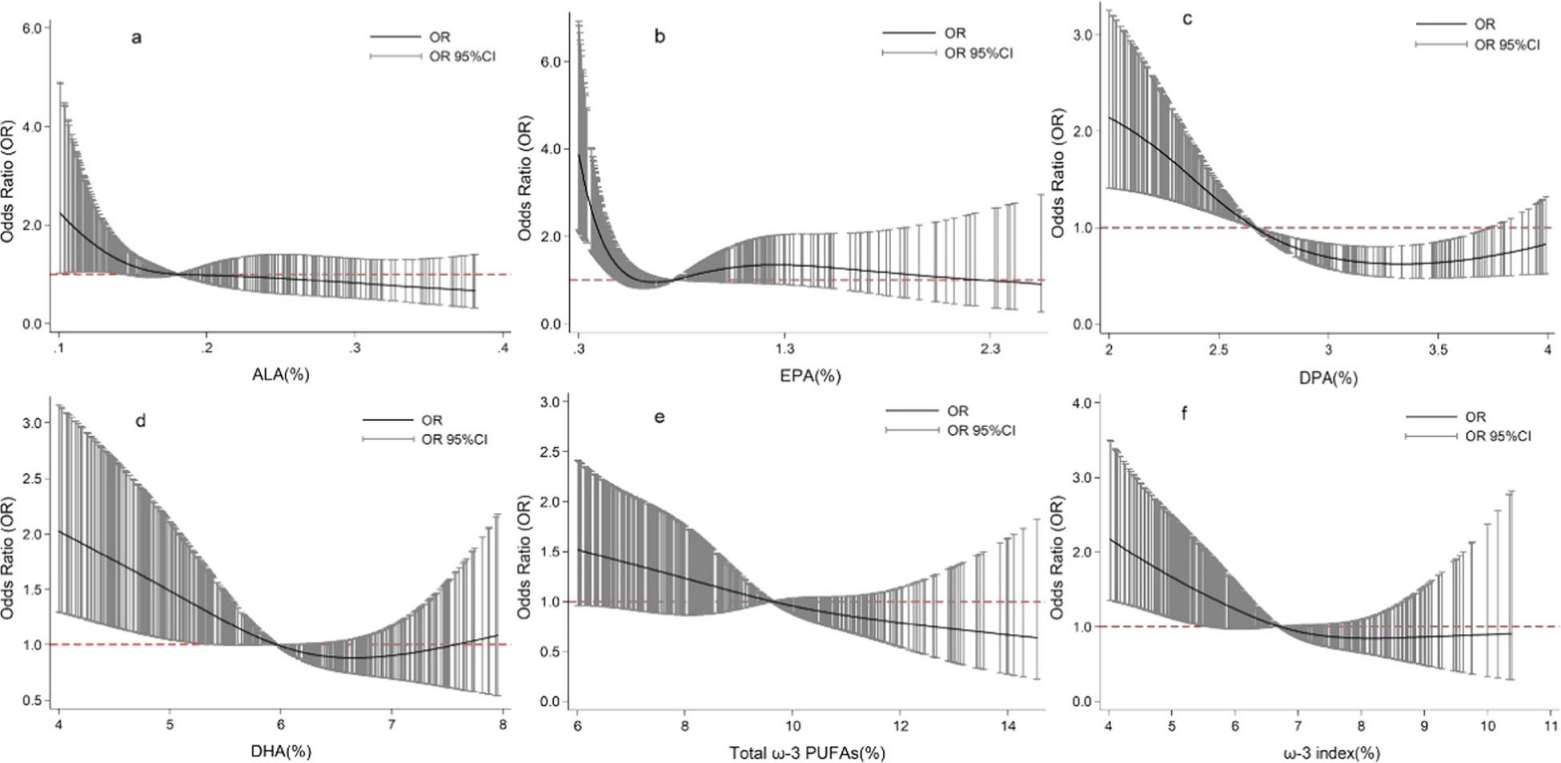
Association between ω-3PUFAs and risk of oral cancer by restricted cubic spline. **a** ALA, **b** EPA, **c** DPA, **d** DHA, **e** Total ω-3PUFAs, **f** ω-3 index. All adjusted for age, sex, education level, BMI, occupation, oral hygiene, smoking, and drinking.

ω-3 PUFAs were presented as median and inter quartile range in Table 2. Erythrocyte ALA, EPA, DPA, DHA, total ω-3 PUFAs, and ω-3 index were significantly lower in the patient group than in the control group. Then, the potential non-linear association between ω-3 PUFAs and the risk of oral cancer were explored using the restricted cubic spline. There is a general pattern that risk of oral cancer decreases with the increase of ALA, DPA, DHA, total ω-3 PUFAs, and ω-3 index. Adverse but non-linear correlation between ω-3 PUFA and oral cancer were observed after adjusted age, sex, education level, BMI, occupation, oral hygiene, smoking, and drinking (Fig. 1).

**Table 2:** Erythrocyte ω-3 PUFAs levels in cancer and control group.

Fatty acid	Control	Case	*P* value
ω-3 PUFA			
ALA (18:3 n-3)	0.18 (0.15–0.24)	0.17 (0.13–0.21)	<0.01
EPA (20:5 n-3)	0.76 (0.55–0.97)	0.56 (0.39–0.89)	<0.01
DPA (C22:5 n-3)	2.66 (2.31–3.09)	2.45 (2.14–2.94)	<0.01
DHA (22:6 n-3)	5.96 (5.22–6.69)	5.47 (4.51–6.43)	<0.01
Total fatty acids			
ω-3 PUFAs	9.61 (8.19–10.80)	8.72 (7.06–10.18)	<0.01
Fatty acid index			
ω-3 index	6.70 (5.96–7.59)	6.17 (5.00–7.20)	<0.01

*Note*: All data were presented as median(inter quartile range).

Next, the crude and adjusted *OR* with 95% CI was calculated using logistic regression. Erythrocyte ω-3 PUFAs were included in unconditional logistic regression model as continuous variable, or as dichotomous variable categorized according to the median of the control group. As shown in Table 3, in the multivariate model which included erythrocyte ω-3 PUFAs as continuous variable. ALA, EPA, DPA, DHA, total ω-3 PUFAs, and ω-3 index were all adversely associated with oral cancer risk, adjusted *OR* of which were 0.51 (95% *CI*: 0.29–0.91), 0.54 (95% *CI*: 0.37–0.79), 0.23 (95% *CI*: 0.09–0.57), 0.19 (95% *CI*: 0.08–0.45), 0.12 (95% *CI*: 0.04–0.32), 0.21 (95% *CI*: 0.09–0.47), respectively. In the multivariate model which included erythrocyte ω-3 PUFAs as dichotomous variable, consistent adverse association was observed for EPA, DHA, total ω-3 PUFAs, and ω-3 index, adjusted *OR* of which were 0.62 (95% *CI*: 0.41–0.92), 0.61 (95% *CI*: 0.41–0.90), 0.66 (95% *CI*: 0.45–0.99), and 0.64 (95% *CI*: 0.43–0.94), respectively.

**Table 3:** Association between ω-3 PUFAs and oral cancer risk by univariable and multivariable logistic regression analysis.

Fatty acid	Control *n* (%)	Case *n* (%)	Univariable	Multivariable
			*OR* (95% *CI*)	Adjusted *OR* (95% *CI*)^a^
ALA (18:3 n-3)				
Continuous variable			0.40 (0.24–0.67)	0.51 (0.29–0.91)
<0.18%	150 (50.00)	149 (63.14)	1.00	1.00
≥0.18%	150 (50.00)	87 (36.86)	0.58 (0.41–0.83)	0.67 (0.45–1.00)
EPA (20:5 n-3)				
Continuous variable			0.41 (0.29–0.58)	0.54 (0.37–0.79)
<0.76%	150 (50.00)	159 (67.37)	1.00	1.00
≥0.76%	150 (50.00)	77 (32.63)	0.48 (0.34–0.69)	0.62 (0.41–0.92)
DPA (C22:5 n-3)				
Continuous variable			0.14 (0.06–0.31)	0.23 (0.09–0.57)
<2.66%	150 (50.00)	150 (63.56)	1.00	1.00
≥2.66%	150 (50.00)	86 (36.44)	0.57 (0.40–0.81)	0.76 (0.51–1.13)
DHA (22:6 n-3)				
Continuous variable			0.14 (0.07–0.30)	0.19 (0.08–0.45)
<5.96%	150 (50.00)	152 (64.41)	1.00	1.00
≥5.96%	150 (50.00)	84 (35.59)	0.55 (0.39–0.78)	0.61 (0.41–0.90)
Total ω-3 PUFAs				
Continuous variable			0.06 (0.02–0.16)	0.12 (0.04–0.32)
<9.61%	150 (50.00)	155 (65.68)	1.00	1.00
≥9.61%	150 (50.00)	81 (34.32)	0.52 (0.37–0.74)	0.66 (0.45–0.99)
ω-3 index				
Continuous variable			0.14 (0.07–0.28)	0.21 (0.09–0.47)
<6.70%	150 (50.00)	150 (63.56)	1.00	1.00
≥6.70%	150 (50.00)	86 (36.44)	0.57 (0.40–0.81)	0.64 (0.43–0.94)

*Note*: All adjusted for age(continuous variable), sex, education level, BMI(continuous variable), oral hygiene, smoking, drinking, diabetes.

Smoking and drinking are important traditional risk factors for oral cancer, which were shown to be associated with oral cancer risk in the current study population (Supplementary Table 1). Additionally, the distribution of ω-3 PUFAs varied between smoker and non-smokers (Supplementary Table 2). So we performed stratified analysis according to smoking and drinking status, to see if there is interaction between erythrocyte ω-3 PUFAs and smoking or drinking in oral cancer. We found that association between EPA and oral cancer risk was only significant in the smoking group, *OR* of which were 0.63 (95% *CI*: 0.39–1.00). Multiplicative interaction was observed between smoking and ALA, EPA, and DHA (*P*_interaction_ < 0.05) (Table 4). Similarly, general multiplicative interaction was also observed between drinking and ALA, EPA, DHA, ω-3 index (*P*_interaction_ < 0.05), and the significant protective effect of ALA, EPA, and DHA were only observed in the non-drinking group, *OR* of which were 0.63 (95% *CI*: 0.41–0.99), 0.57 (95% *CI*: 0.36–0.88), 0.64 (95% *CI*: 0.41–0.98), respectively (Table 5).

**Table 4:** Association between ω-3 PUFAs and oral cancer stratified by smoking.

Fatty acid	Non-smoking	Smoking	a *OR* (95% *CI*) for interaction	*P* interaction
	*OR* (95% *CI*)	*OR* (95% *CI*)		
ALA (18:3 n-3)				
<0.18%	1.00	1.00	0.62 (0.39–0.98)	0.04
≥0.18%	0.66 (0.41–1.05)	0.58 (0.25–1.32)		
EPA (20:5 n-3)				
<0.76%	1.00	1.00	0.57 (0.36–0.88)	0.01
≥0.76%	0.63 (0.39–1.00)	0.55 (0.24–1.27)		
DHA (22:6 n-3)				
<5.96%	1.00	1.00	0.60 (0.39–0.93)	0.02
≥5.96%	0.67 (0.42–1.06)	0.47 (0.21–1.05)		
Total ω-3 PUFAs				
<9.61%	1.00	1.00	0.67 (0.43–1.04)	0.08
≥9.61%	0.74 (0.47–1.18)	0.44 (0.20–0.99)		
ω-3 index				
<6.70%	1.00	1.00	0.65 (0.42–1.02)	0.06
≥6.70%	0.74 (0.46–1.17)	0.39 (0.17–0.89)		

*Note*: All adjusted for age(continuous variable), sex, education level, BMI(continuous variable), oral hygiene, drinking, diabetes.

**Table 5:** Association between ω-3 PUFAs and oral cancer stratified by drinking.

Fatty acid	Non-drinking	Drinking	a *OR* (95% *CI*) for interaction	*P* interaction
	*OR* (95% *CI*)	*OR* (95% *CI*)		
ALA (18:3 n-3)				
<0.18%	1.00	1.00	0.60 (0.39–0.93)	0.02
≥0.18%	0.63 (0.41–0.99)	0.64 (0.22–1.89)		
EPA (20:5 n-3)				
<0.76%	1.00	1.00	0.54 (0.35–0.83)	0.01
≥0.76%	0.57 (0.36–0.88)	1.01 (0.36–2.89)		
DHA (22:6 n-3)				
<5.96%	1.00	1.00	0.60 (0.39–0.92)	0.02
≥5.96%	0.64 (0.41–0.98)	0.56 (0.20–1.56)		
Total ω-3 PUFAs				
<9.61%	1.00	1.00	0.64 (0.42–0.97)	0.04
≥9.61%	0.67 (0.43–1.03)	0.74 (0.27–2.04)		
ω-3 index				
<6.70%	1.00	1.00	0.62 (0.40–0.94)	0.02
≥6.70%	0.65 (0.42–1.01)	0.69 (0.24–1.96)		

*Note*: All adjusted for age(continuous variable), sex, education level, BMI(continuous variable), oral hygiene, smoking, diabetes.

## Discussion

The potential role of ω-3 PUFAs in carcinogenesis has long been controversial. A number of studies have reported adverse association between blood ω-3 PUFAs and cancer risk. Matejcic et al. conducted a cohort study to explore the association between plasma fatty acids and the risk of pancreatic cancer, they found that plasma ω-3 polyunsaturated fatty acid α-linolenic acid and docosapentaenoic acid was negatively correlated with pancreatic cancer^19^. Kim et al. performed a prospective systematic review and meta-analysis, results of which showed that the combined RR of the highest and lowest levels of ω-3 PUFAs was 0.79 (95% CI: 0.64–0.98) in colorectal cancer^13^. Meanwhile, another meta-analysis noted a significant adverse association between high serum DPA and total prostate cancer risk (RR: 0.756; 95% CI: 0.599, 0.955)^20^. However, there are also evidence supporting that plasma ω-3 PUFAs are not associated with cancer risk^21,22^ or even positively associated with cancer risk^23^. Bassett et al. performed a case-cohort analysis with 1717 men and 464 prostate cancer cases to investigate the association between fatty acids in plasma and prostate cancer risk, and no association was found. And not coincidentally, Chaje’s et al. found that plasma phospholipid fatty acid EPA, DPA, DHA had no association with gastric cancer risk^24^. Brasky et al. conducted a case-cohort study which included 2273 men and explored the association between plasma long-chain ω-3 PUFAs and the risk of prostate cancer. They noted that long-chain ω-3 PUFAs (DPA and DHA) were positively correlated with the risk of prostate cancer^23^. In our study, we observed that erythrocyte ALA, EPA, DPA, DHA, total ω-3 PUFAs, and ω-3 index was significantly higher in control participants than oral cancer patients. Non-linear association was observed between ALA, EPA, DHA, total ω-3 PUFAs, ω-3 index, and oral cancer. The risk of oral cancer decreased with the increase of ALA, DPA, DHA, total ω-3 PUFAs, and ω-3 index. To the best of our knowledge, reports of the association between serum or erythrocyte membrane ω-3 PUFAs and oral cancer are rare. Our findings are consistent with two HNC studies which involved dietary fatty acid intake. One study from Italy and Switzerland illustrated that higher intake of ω-PUFAs was associated with the decrease of oral cancer risk, the multivariate odds ratios (OR) for the highest quintile of ω-3 PUFAs compared to the lowest one was 0.5^25^. The other study from the US also reported higher intake of dietary ω-PUFAs was associated with decreased HNC risk^11^.

In addition to epidemiological studies, some in vitro and in vivo experiments have also confirmed the protective effects of ω-PUFAs in tumorigenesis. A study explored the effects of ω-PUFAs on tumorigenic keratinocyte lines—epidermal (facial epidermis) and oral (tongue) noted that ω-PUFAs can activate ERK1/2 pathway through EGFR, causing both reduced proliferation and apoptosis^26^. Another in vivo experiment noted that ω-3 PUFAs play an antioxidant role in immortalized mouse Schwann cells through nuclear factor-related factor 2 pathway^27^. Furthermore, studies exploring the relationship between plasma PUFAs and biomarkers of oxidative stress of breast cancer found that ω-PUFAs can modulate the critical processes of breast carcinogenesis with oxidation and inflammation^28^.

Moreover, we observed general interaction between ω-3 PUFAs and traditional risk factors such as smoking and drinking. Erythrocyte EPA, DHA, and ω-3 index were lower in smokers compared with non-smokers. This is consistent with reports by Zehr et al.^29^. Possible explanation is that the general dietary intake of nutrients in smokers is lower than that of non-smokers^30^. We also found that the adverse correlation between EPA and oral cancer was only significant in the non-smoking group, while the adverse correlation of ALA, EPA, and DHA were only significant in the non-drinking group, which may be attributed to the fact that the anti-inflammatory and protective effect of ω-3 PUFAs was partially counteracted by the damage caused by smoking or drinking^31^. Future studies are needed to better understand the mechanism underlying the interaction between ω-3 PUFAs, smoking and drinking.

There are several limitations for this study. Firstly, this is a case-control study, since there is a long latency period which means that exposure data must be available for a period of several decades or more in order to identify true overall risks. The erythrocyte fatty acids data was not collected prospectively. Erythrocyte fatty acids may be affected by changes in dietary patterns or other behaviors due to symptoms from the disease. Case-control studies can be affected by reverse causality and cannot establish temporality. Thus, the associations observed between erythrocyte fatty acids and oral cancer should be interpreted cautiously. Secondly, as for the control of confounding factors, age, sex, ethnic group, marital status, education level, origin, family history of cancer, BMI, occupation, smoking, drinking were adjusted. But we did not collect information of medication affecting fatty acid levels, and this may cause potential confounding. Lastly, the sample size of this study is limited, further study can increase the sample size to confirm the current findings.

## Conclusion

The risk of oral cancer decreased with the increase of ω-3 PUFAs. There were adverse association between erythrocyte EPA, DHA, total ω-3 PUFAs, ω-3 index, and oral cancer risk. Additionally, multiplicative interactions between ω-3 PUFAs and other behavior factors such as smoking and drinking were observed. The protective effect of ω-3 PUFAs may be more significant in the non-smoking or non-drinking population. Future prospective research are needed to confirm the current findings.

## Supplementary information

Supplemental tables
