# Supplementary material for: Erythrocyte ω-3 polyunsaturated fatty acids are inversely associated with the risk of oral cancer: a case-control study
Source: Nutr Diabetes. 2020 Sep 28;10:35. doi: 10.1038/s41387-020-00140-1 (PMC7522234; doi:10.1038/s41387-020-00140-1)
Supplement: Supplementary file 1 — Supplemental tables [file 41387_2020_140_MOESM1_ESM.docx]

| Supplemental table 1 Risk factors of oral cancer by univariate and multivariate logistic regression analysis | | | | | |
| --- | --- | --- | --- | --- | --- |
| variables | Control N(%) | Case N(%) | Univariate |  | Multivariate |
|  |  |  | *OR(95% CI)* |  | adjusted *OR(95% CI)* |
| Smoking ^a^ |  |  |  |  |  |
| no | 241(80.33) | 152(64.41) | 1.00 |  | 1.00 |
| yes | 59(19.67) | 84(35.59) | 2.26(1.53-3.33) |  | 1.92(1.02-3.61) |
| Alcohol consumption ^b^ |  |  |  |  |  |
| no | 259(86.33) | 175(74.15) | 1.00 |  | 1.00 |
| yes | 41(13.67) | 61(25.85) | 2.20(1.42-3.42) |  | 1.54(0.85-2.77) |
| Note: a refers to adjusted for age(continuous variable), sex, education level, BMI(continuous variable), oral hygiene, drinking, diabetes; b refers to adjusted for age(continuous variable), sex, education level, BMI(continuous variable), oral hygiene, smoking, diabetes | | | | | |

| Supplementary table 2 Erythrocyte ω-3 PUFAs distribution stratified by smoking/drinking | | | | | | | | | |
| --- | --- | --- | --- | --- | --- | --- | --- | --- | --- |
| Fatty acid | Non-smoker | Smoker | *P* |  | Non-drinker | | Drinker | | *P* |
| ω-3 PUFAs |  |  |  |  |  |  | |  | |
| ALA (18:3 n-3) | 0.18(0.14-0.22) | 0.17(0.13-0.21) | 0.05 |  | 0.18(0.14-0.22) | 0.17(0.14-0.21) | | 0.27 | |
| EPA (20:5 n-3) | 0.72(0.49-0.97) | 0.61(0.42-0.88) | 0.01 |  | 0.71(0.47-0.95) | 0.62(0.43-0.90) | | 0.11 | |
| DPA(C22:5 n-3) | 2.60(2.19-3.04) | 2.54(2.20-3.01) | 0.97 |  | 2.60(2.20-3.07) | 2.52(2.20-3.00) | | 0.58 | |
| DHA (22:6 n-3) | 5.88(4.94-6.66) | 5.55(4.67-6.33) | 0.02 |  | 5.83(4.81-6.56) | 5.74(4.90-6.44) | | 0.48 | |
| Total fatty acids |  |  |  |  |  |  | |  | |
| ω-3 PUFAs | 9.37(7.81-10.56) | 8.81(7.43-10.39) | 0.16 |  | 9.32(7.72-10.55) | 8.83(7.68-10.38) | | 0.42 | |
| Fatty acid indexes |  |  |  |  |  |  | |  | |
| ω-3 index | 6.64(5.56-7.57) | 6.35(5.25-7.08) | 0.02 |  | 6.55(5.47-7.46) | 6.51(5.53-7.34) | | 0.49 | |
| Note: all data was show as median(InterQuartile Range) | | | | | | | | | |
